# Supplementary material for: Dietary Management of Chronic Kidney Disease and Secondary Hyperoxaluria in Patients with Short Bowel Syndrome and Type 3 Intestinal Failure
Source: Nutrients. 2022 Apr 14;14(8):1646. doi: 10.3390/nu14081646 (PMC9030588; doi:10.3390/nu14081646)
Supplement: Supplementary file 1 [file nutrients-14-01646-s001.zip › nutrients-1639311-Figure S1-PRISMA diagram.pdf]

PRISMA 2020 flow diagram for new systematic reviews which included searches of databases and registers only

Figure S1 – PRISMA Diagram

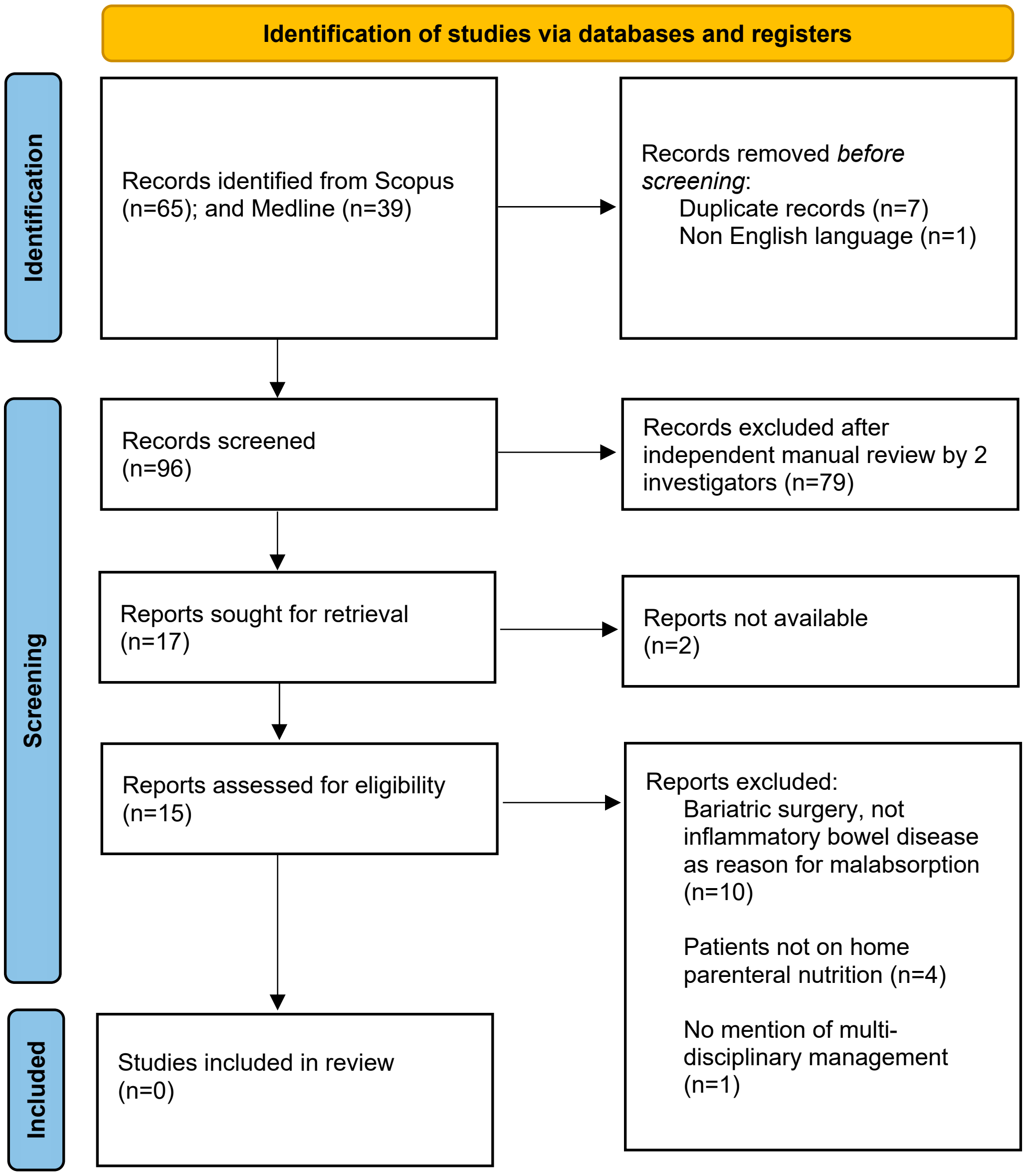

Adapted from: Page MJ, McKenzie JE, Bossuyt PM, Boutron I, Hoffmann TC, Mulrow CD, et al. The PRISMA 2020 statement: an updated guideline for reporting systematic reviews. BMJ 2021;372:n71. doi: 10.1136/bmj.n71
